# Supplementary figures and images for: Dynamic Na+/H+ exchanger 1 (NHE1) – calmodulin complexes of varying stoichiometry and structure regulate Ca2+-dependent NHE1 activation
Source: eLife. 2021 Mar 3;10:e60889. doi: 10.7554/eLife.60889 (PMC8009664; doi:10.7554/eLife.60889)

## Slide 1
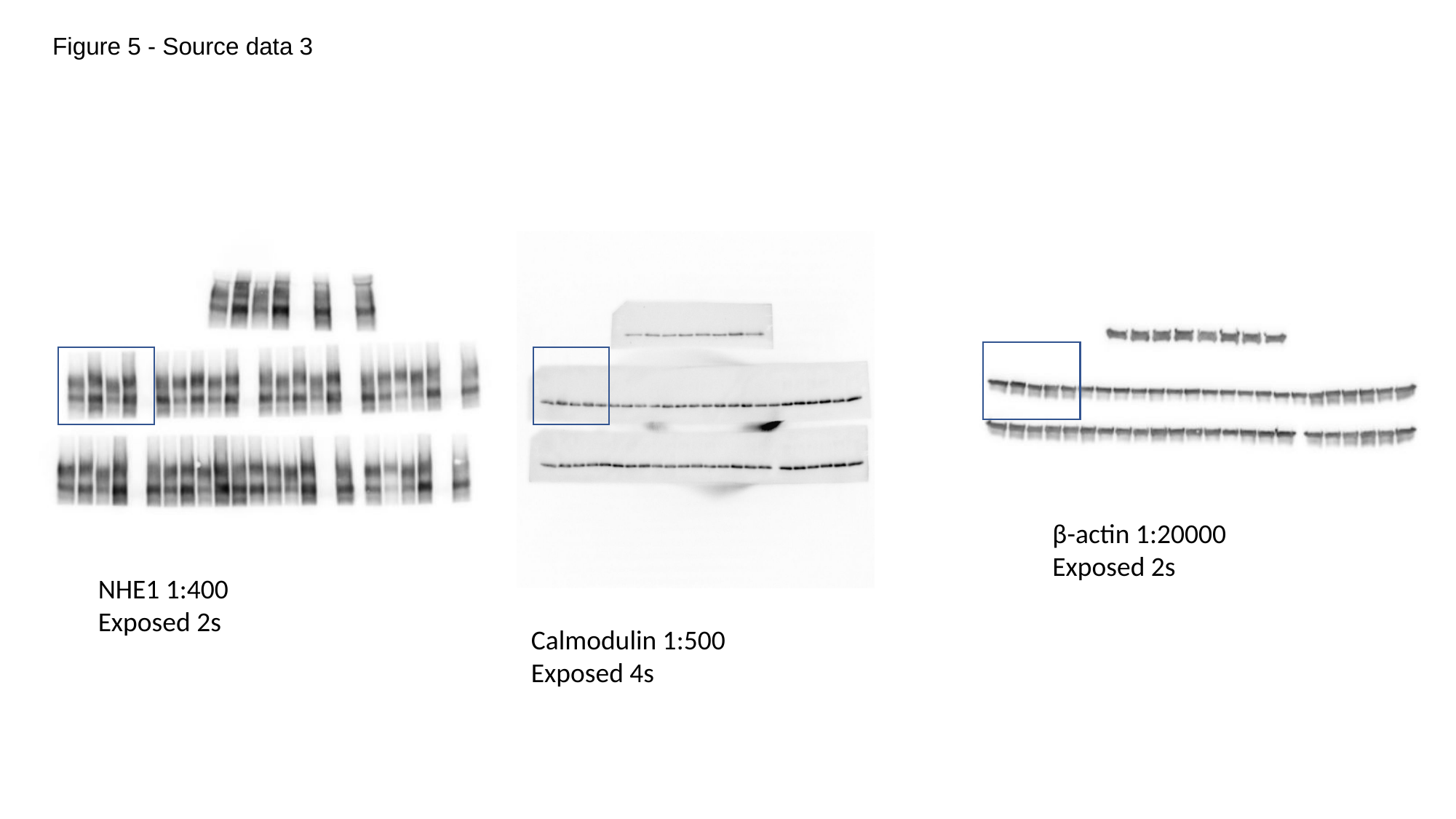

# Figure 5 - Source data 3
β-actin 1:20000
Exposed 2s
NHE1 1:400
Exposed 2s
Calmodulin 1:500
Exposed 4s

Supplement: Figure 5—source data 3. [file elife-60889-fig5-data3.pptx]
