# Supplementary material for: Dynamic Na+/H+ exchanger 1 (NHE1) – calmodulin complexes of varying stoichiometry and structure regulate Ca2+-dependent NHE1 activation
Source: eLife. 2021 Mar 3;10:e60889. doi: 10.7554/eLife.60889 (PMC8009664; doi:10.7554/eLife.60889)
Supplement: Figure 6—source data 3. [file elife-60889-fig6-data3.pptx]

## Slide 1
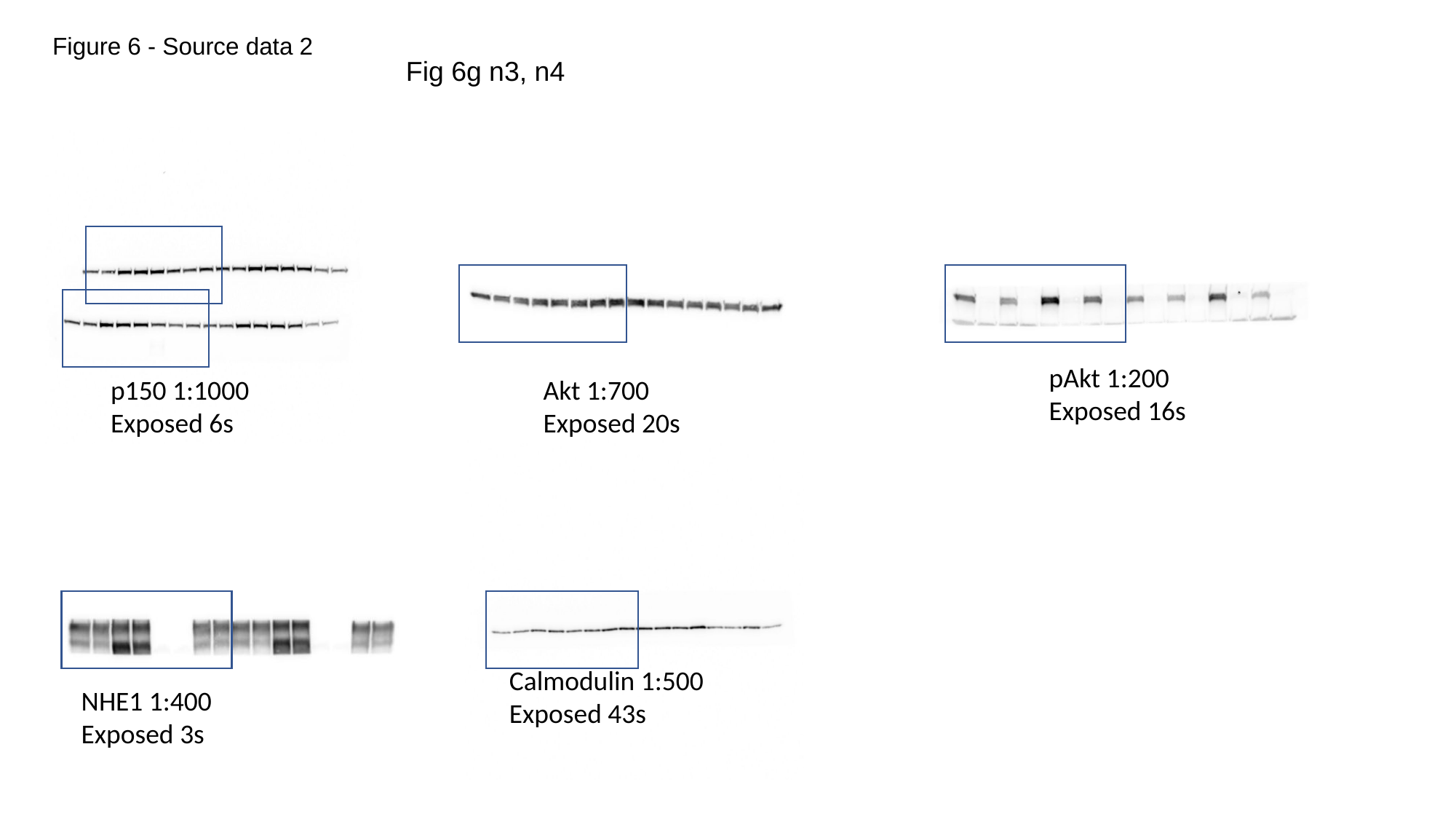

# Fig 6g n3, n4
Figure 6 - Source data 2
pAkt 1:200
Exposed 16s
Akt 1:700
Exposed 20s
p150 1:1000
Exposed 6s
Calmodulin 1:500
Exposed 43s
NHE1 1:400
Exposed 3s

## Slide 2
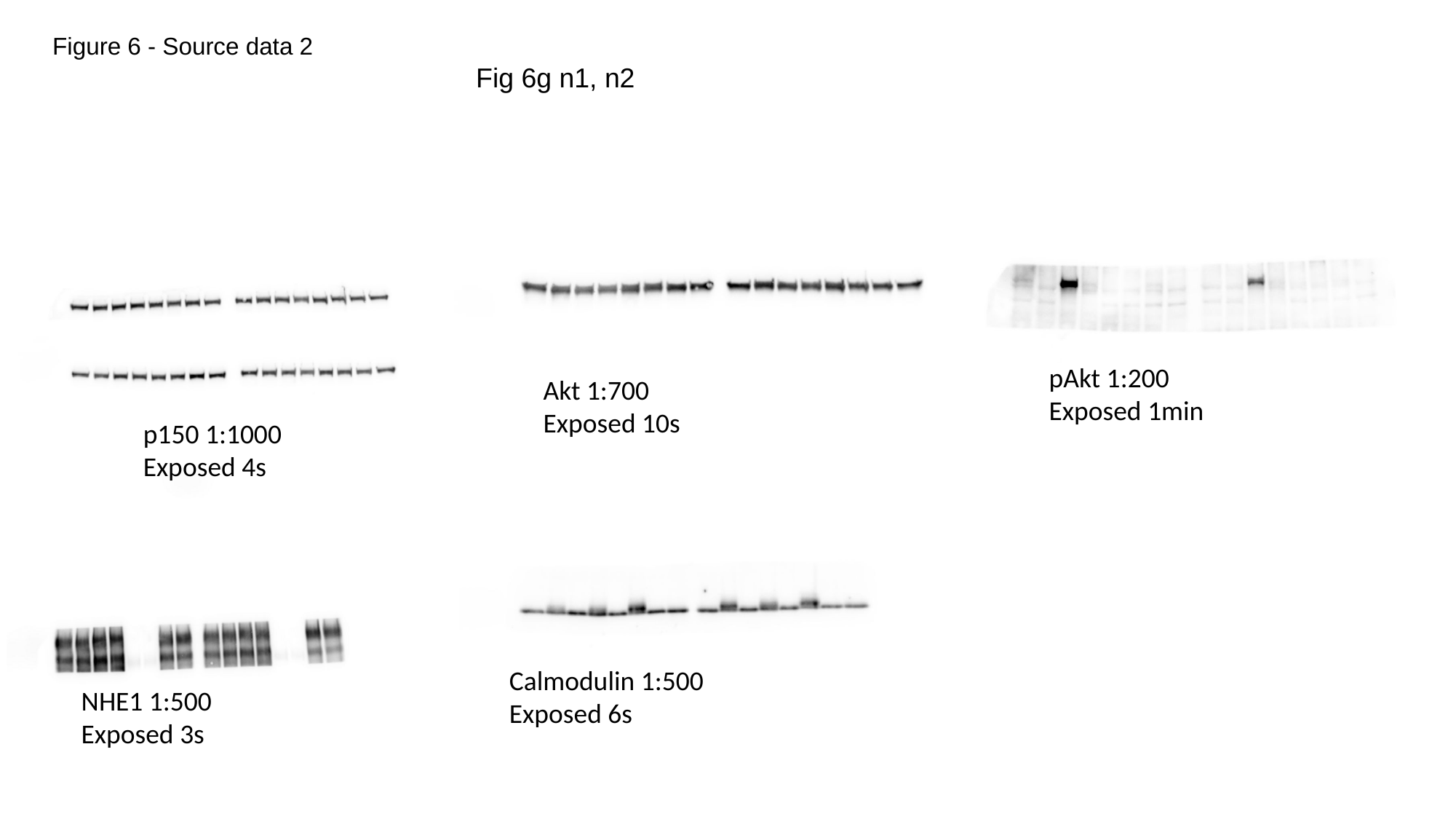

# Fig 6g n1, n2
Figure 6 - Source data 2
pAkt 1:200
Exposed 1min
Akt 1:700
Exposed 10s
p150 1:1000
Exposed 4s
Calmodulin 1:500
Exposed 6s
NHE1 1:500
Exposed 3s
