# Supplementary material for: Dynamic Na+/H+ exchanger 1 (NHE1) – calmodulin complexes of varying stoichiometry and structure regulate Ca2+-dependent NHE1 activation
Source: eLife. 2021 Mar 3;10:e60889. doi: 10.7554/eLife.60889 (PMC8009664; doi:10.7554/eLife.60889)
Supplement: Transparent reporting form [file elife-60889-transrepform.pdf]

eLIFE
1st Floor
24 Hills Road
Cambridge CB2 1JP, UK
P 01223 855340
W elifesciences.org
T @elife

eLife's transparent reporting form

We encourage authors to provide detailed information *within their submission* to facilitate the interpretation and replication of experiments. Authors can upload supporting documentation to indicate the use of appropriate reporting guidelines for health-related research (see EQUATOR Network), life science research (see the BioSharing Information Resource), or the ARRIVE guidelines for reporting work involving animal research. Where applicable, authors should refer to any relevant reporting standards documents in this form.

If you have any questions, please consult our Journal Policies and/or contact us: editorial@elifesciences.org.

Sample-size estimation

- You should state whether an appropriate sample size was computed when the study was being designed
- You should state the statistical method of sample size computation and any required assumptions
- If no explicit power analysis was used, you should describe how you decided what sample (replicate) size (number) to use

Please outline where this information can be found within the submission (e.g., sections or figure legends), or explain why this information doesn't apply to your submission:

Sample size was estimated based on our team's previous experience with the types of experiments conducted. Calculations were not included in the submission.

Replicates

- You should report how often each experiment was performed
- You should include a definition of biological versus technical replication
- The data obtained should be provided and sufficient information should be provided to indicate the number of independent biological and/or technical replicates
- If you encountered any outliers, you should describe how these were handled
- Criteria for exclusion/inclusion of data should be clearly stated
- High-throughput sequence data should be uploaded before submission, with a private link for reviewers provided (these are available from both GEO and ArrayExpress)

Please outline where this information can be found within the submission (e.g., sections or figure legends), or explain why this information doesn't apply to your submission:

All information pertaining to replicates is provided in the figure legends as, e.g. (n=6), where n refers to the number of biological replicates, defined as independently conducted experiments on different passages of cells/different experiment days. Outliers were only defined as such quantitatively using Dixon's Q test and $p < 0.05$.
For pH experiments carried out with the FluoStar plate reader, traces with obviously unacceptable signal to noise ratio and traces showing recovery during the Na+ free step were excluded to ensure that only full initial NHE1 activity was recorded. No high-throughput sequence data is used in the manuscript.

Statistical reporting

- Statistical analysis methods should be described and justified
- Raw data should be presented in figures whenever informative to do so (typically when N per group is less than 10)
- For each experiment, you should identify the statistical tests used, exact values of N, definitions of center, methods of multiple test correction, and dispersion and precision measures (e.g., mean, median, SD, SEM, confidence intervals; and, for the major substantive results, a measure of effect size (e.g., Pearson's r, Cohen's d)
- Report exact p-values wherever possible alongside the summary statistics and 95% confidence intervals. These should be reported for all key questions and not only when the p-value is less than 0.05.

Please outline where this information can be found within the submission (e.g., sections or figure legends), or explain why this information doesn't apply to your submission:

Details of all statistical methods used are given in the Materials and Methods section under "Statistical analysis", and other information for individual experiments are given in figure legends. For cell biological experiments, raw data and/or all individual data points for summarized data are provided in all figures. The statistical analysis of the solved structure is attached as obtained from the wwPDB validation server. Please note that the chemical shift list 2 and 3 were linked to the wrong molecule by wwPDB and we are waiting for a correction from the staff of wwPDB.

(For large datasets, or papers with a very large number of statistical tests, you may upload a single table file with tests, Ns, etc., with reference to sections in the manuscript.)

Group allocation

- Indicate how samples were allocated into experimental groups (in the case of clinical studies, please specify allocation to treatment method); if randomization was used, please also state if restricted randomization was applied
- Indicate if masking was used during group allocation, data collection and/or data analysis

Please outline where this information can be found within the submission (e.g., sections or figure legends), or explain why this information doesn't apply to your submission:

eLife Sciences Publications, Ltd is a limited liability non-profit non-stock corporation incorporated in the State of Delaware, USA, with company number 5030732, and is registered in the UK with company number FC030576 and branch number BR015634 at the address 1st Floor, 24 Hills Road, Cambridge CB2 1JP | August 2014

No clinical data or other studies requiring randomization were employed. Quantification of PLA data was carried out blinded and using image analysis software with identical methods across experimental groups.

Additional data files ("source data")

- We encourage you to upload relevant additional data files, such as numerical data that are represented as a graph in a figure, or as a summary table
- Where provided, these should be in the most useful format, and they can be uploaded as "Source data" files linked to a main figure or table
- Include model definition files including the full list of parameters used
- Include code used for data analysis (e.g., R, MatLab)
- Avoid stating that data files are "available upon request"

Please indicate the figures or tables for which source data files have been provided:

The raw data of all ITC experiments is provided in numerical form and the individual measurements of each triplicate are given in the folder ITC_Source-data. NMR data files are available upon request.
